# *Gonatopusjaliscanus* sp. n., a new Pincer wasp from Jalisco, Mexico (Hymenoptera, Dryinidae)

**DOI:** 10.3897/zookeys.818.30974

**Published:** 2019-01-17

**Authors:** Stefano Speranza, Massimo Olmi, Adalgisa Guglielmino, Mario Contarini

**Affiliations:** 1 Department of Agriculture and Forest Sciences (DAFNE), University of Tuscia, Viterbo, Italy University of Tuscia Viterbo Italy; 2 Tropical Entomology Research Center, Viterbo, Italy Tropical Entomology Research Center Viterbo Italy

**Keywords:** Chrysidoidea, Gonatopodinae, keys, taxonomy

## Abstract

A new species of *Gonatopus* Ljungh, 1810, *G.jaliscanus***sp. n.**, from Jalisco, Mexico, is described and illustrated. In the Neotropical region, *G.jaliscanus* is similar to *G.forestalis* Olmi, 1998, but it is distinguished by the black mesosoma (except prothorax, mesoscutum, and mesoscutellum that are yellow), and the metapostnotum being granulated and not rugose; in *G.forestalis* the mesosoma is completely black and the metapostnotum is granulated and strongly rugose. In the Nearctic region, the new species is morphologically similar to *G.curriei* Krombein, 1962, but it is distinguished by the dull and granulated metapostonotum; in *G.curriei* the metapostnotum is shiny and unsculptured. The new species belongs to *Gonatopus* group 7. The keys to the females of the Nearctic and Neotropical species of this group are modified to include the new taxon.

## Introduction

Dryinidae (Hymenoptera: Chrysidoidea) are parasitoids and often also predators of leafhoppers, planthoppers and treehoppers (Hemiptera, Auchenorrhyncha) (Guglielmino et al. 2013). They comprise 16 subfamilies, 50 genera and more than 1800 species worldwide (Olmi and Xu 2015, Tribull 2015).

Jalisco is a state of Mexico situated in a transition area between the Nearctic and Neotropical regions. Species of Dryinidae collected in this state can belong to either region, so for the identification, researchers have to check the keys of both zoogeographical regions. Dryinidae of the Nearctic and Neotropical regions were studied mainly respectively by Olmi (1984) and Olmi and Virla (2014).

In the Nearctic and Neotropical regions respectively, the genus *Gonatopus* Ljungh, includes 51 (Olmi 1984, 1987, 1992, 1993, 1995, 2003, 2010, Olmi and Guglielmino 2013) and 127 species (Martins and Domahovski 2017a, b, Martins et al. 2015a, b, Martins and Krinski 2016, Olmi and Guglielmino 2016, Olmi and Virla 2014). For its part, Mexico is inhabited by 135 species of Dryinidae and 25 of *Gonatopus* (Moya-Raygoza and Olmi 2010, Becerra-Chiron et al. 2017). In 2017 the authors examined a species of *Gonatopus* collected in Jalisco, Mexico, which is described as new below.

## Materials and methods

The description follows the terminology used by Guglielmino et al. (2016, 2018a, b) and Olmi and Virla (2014). The measurements reported are relative, except for the total length (head to abdominal tip, without the antennae), which is expressed in millimetres. In the descriptions POL is the distance between the inner edges of the lateral ocelli; OL is the distance between the inner edges of a lateral ocellus and the median ocellus; OOL is the distance from the outer edge of a lateral ocellus to the compound eye.

The term “metapectal-propodeal complex” is here used in the sense of Kawada et al. (2015). It corresponds to the term “metathorax + propodeum” sensu Olmi (1984) and Olmi and Virla (2014). In apterous Gonatopodinae the terms “anterior surface of metathorax + propodeum” and “posterior surface of metathorax + propodeum”, sensu Olmi (1984, 1994), Olmi and Virla (2014), Olmi and Xu (2015) and Xu et al. (2013), correspond here respectively to “metapostonotum” and “first abdominal tergum”, sensu Kawada et al. (2015).

The types of all Nearctic and Neotropical species of *Gonatopus* were examined. The material studied in this paper will be deposited in the National Museum of Natural History, Washington, DC, USA (**USNM**).

The description of the new species is based on the study of only a single specimen. The authors are aware that descriptions of new taxa should normally be based on more individuals. However, Dryinidae are so rare that it is uncommon to collect more than one specimen of each species. In addition, on the basis of the experience and knowledge of the authors, the new species is sufficiently delimited by unique characters to justify its description.

## Results

### Genus Gonatopus

Taxon classificationAnimaliaAlismatalesAraceae

Ljungh, 1810

Gonatopus Ljungh, 1810: 161. Type species: Gonatopusformicarius Ljungh, 1810, by monotypy.

#### Diagnosis of the genus.

Female: Apterous or less frequently macropterous; palpal formula 3/2, 4/2, 4/3, 5/2, 5/3, or 6/3; pronotum crossed or not by transverse furrow; enlarged claw with distal apex pointed and with one large or small subapical tooth (occasionally subapical tooth absent, then enlarged claw with distal group of lamellae); in fully winged forms, segment 5 of protarsus with more than 20 lamellae; tibial spurs 1/0/1. Male: Macropterous; occipital carina absent or incomplete (in this last case, present behind and shortly on sides of posterior ocelli); occiput concave; temple present; palpal formula 3/2, 4/2, 4/3, 5/2, 5/3, or 6/3; tibial spurs 1/1/2.

### Gonatopus jaliscanus sp. n.

Taxon classificationAnimaliaAlismatalesAraceae

http://zoobank.org/FEDDFE9E-CD12-4AB7-BC5E-7393473EA1A0

Figs 1 , 2

#### Diagnosis.

**Figure 1.**
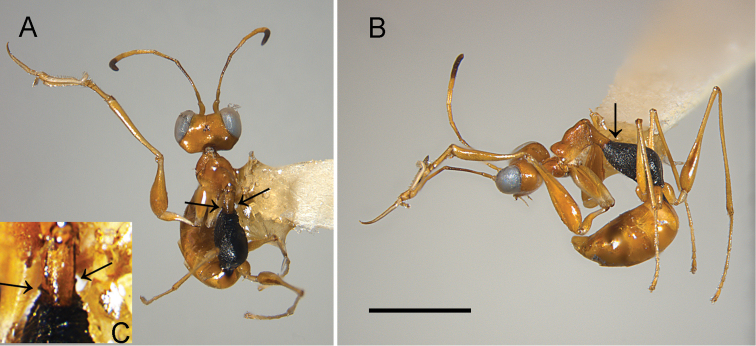
*Gonatopusjaliscanus* sp. n., female holotype: **A** habitus in dorsal view **B** habitus in lateral view **C** magnification of mesoscutum. Arrows indicate lateral apophyses of mesoscutum (**A, C**), and metanotum (**B**). Scale bars: 1.4 mm (**A, B**); 0.6 mm (**C**).

**Figure 2.**
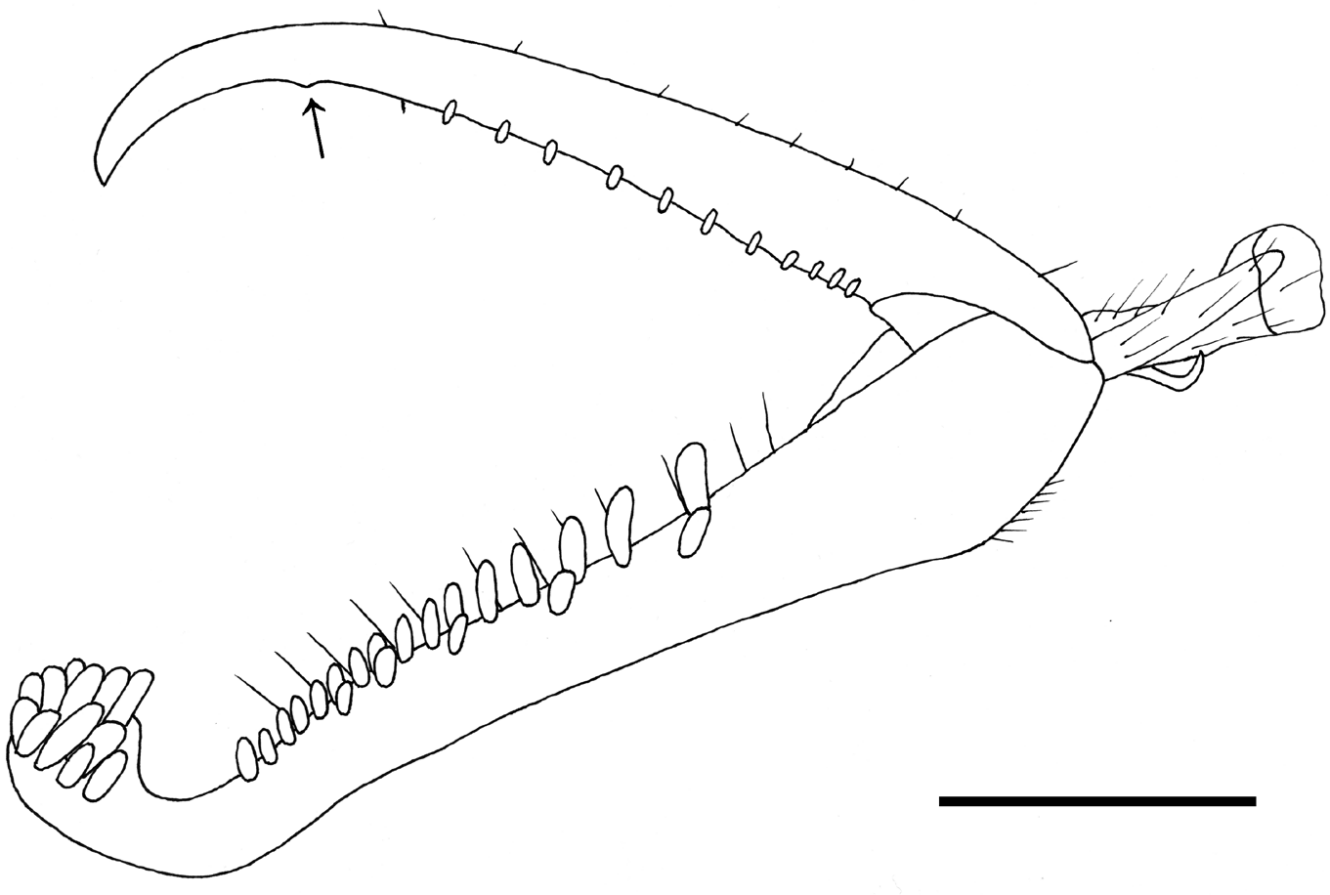
*Gonatopusjaliscanus* sp. n., female holotype: chela. Arrow indicates the enlarged claw subapical tooth. Scale bar 0.19 mm.

Female apterous, with mesosoma black, except prothorax, mesoscutum and mesoscutellum yellow; palpal formula 6/3; pronotum crossed by strong transverse furrow (Fig. 1B); stalk between pronotum and metapectal-propodeal complex about as long as disc of pronotum; mesoscutum laterally with two pointed apophyses (Fig. 1A); meso-metapleural suture obsolete; mesopleuron and metapleuron granulated, not transversely striate; metapostnotum granulated; first abdominal tergum transversely striate; protarsomere 1 shorter than 4; enlarged claw with one small subapical tooth (Fig. 2).

#### Description.

**Female**. Apterous (Fig. 1). Length 3.4 mm. Head, prothorax, mesoscutum, mesoscutellum, metasoma and legs testaceous. Antenna testaceous, except antennomeres 8–10 brown. Metanotum, metapectal-propodeal complex, mesopleuron, metapleuron and petiole black. Antenna clavate. Antennomeres in following proportions: 8:6:21:12:10:8:7:5:5:6. Head excavated, shiny, very weakly granulated. Frontal line complete. Occipital carina absent. POL = 2; OL = 2; OOL = 8. Greatest breadth of lateral ocelli shorter than POL (1:2). Palpal formula 6/3. Pronotum shiny, unsculptured, crossed by strong transverse furrow (Fig. 1B). Mesoscutum laterally with two pointed apophyses (Fig. 1A). Metanotum inclined, not transversely striate, not hollow behind mesoscutellum (Fig. 1B). Metapectal-propodeal complex with metapostnotum dull, granulated. First abdominal tergum granulated and transversely striated. Mesopleuron and metapleuron dull, granulated, not transversely striated. Meso-metapleural suture obsolete. Protarsomeres in following proportions: 13:3:5:20:30. Protarsomeres 2 and 3 produced into hooks. Enlarged claw (Fig. 2) with one small subapical tooth and eleven peg-like hairs, in addition to one bristle. Protarsomere 5 (Fig. 2) with two rows of 16 + 5 lamellae extending beyond 0.5 length of protarsomere and distal apex provided with about 17 lamellae. Tibial spurs 1/0/1.

**Male**. Unknown.

#### Material examined.

**Holotype**: female, MEXICO: Jalisco, 8.3 mi. S Autlan, Hwy 80, 5000’, 8/vii/1984, on Oaks, Oak Forest, JB Woolley (TAMU (to be deposited in USNM)).

#### Hosts.

Unknown.

#### Distribution.

Mexico (Jalisco).

#### Etymology.

The species is named after the state of Jalisco, where the holotype was collected.

#### Remarks.

The female of the new species is apterous, with pronotum crossed by a strong transverse furrow (Fig. 1B), the enlarged claw provided of one small subapical tooth (Fig. 2) and the palpal formula 6/3. Because of these characters, *G.jaliscanus* belongs to group 7 of *Gonatopus*, according to the systematics proposed by Olmi and Virla (2014). In this species, the head is excavated, the labial palpus is 3-segmented, the mesoscutum has two lateral pointed apophyses situated in the stalk between pronotum and metapectal-propodeal complex (Fig. 1A), the metanotum is sloping anteriorly (Fig. 1B), the meso-metapleural suture is obsolete, the first abdominal tergum is completely transversely striate, the protarsomere 1 is shorter than protarsomere 4 (Fig. 1A). In the Nearctic region, there is only one species of *Gonatopus* group 7 with the above characters: *G.curriei* Krombein, 1962. The new species can be included in the key to the females of the Nearctic species of *Gonatopus* group 7 presented by Olmi (1984) by replacing couplet 30 as follows:

**Table:** 

30	Protarsomere 4 slightly shorter than 1	***G.argyrias* (Perkins)**
–	Protarsomere 4 longer than 1	**30**’
30’	Metapostnotum shiny, unsculptured	***G.curriei* Krombein**
–	Metapostnotum dull, granulated	***G.jaliscanus* sp. n.**

In the Neotropical region, *G.jaliscanus* is similar to *G.forestalis* Olmi, 1998. The new species can be included in the key to the females of the Neotropical species of *Gonatopus* group 7 presented by Olmi and Virla (2014) by replacing couplet 51 as follows:

**Table:** 

51	Mesoscutum laterally with two strong pointed apophyses (Fig. 1A)	**51**’
–	Mesoscutum laterally without pointed apophyses	**52**
51’	Mesosoma totally black; metapostnotum granulated and strongly rugose	***G.forestalis* Olmi**
–	Mesosoma black, except yellow prothorax, mesoscutum and mesoscutellum (Fig. 1); metapostnotum granulated but not rugose	***G.jaliscanus* sp. n.**

## Conclusions

Species of *Gonatopus* from Mexico are known mainly through the monographs on Dryinidae of the Nearctic (Olmi 1984) and Neotropical regions (Olmi and Virla 2014), the checklist of Moya-Raygoza and Olmi (2010) and the paper of Becerra-Chiron et al. (2017) totalling 25 species of *Gonatopus* from the country. Following the above description of *G.jaliscanus*, the *Gonatopus* species known from Mexico now number 26.

In Brazil, there are 31 described *Gonatopus* species (Olmi and Virla 2014, Martins et al. 2015a, b, Martins and Krinski 2016, Martins and Domahovski 2017a, b); in Costa Rica 22 (Olmi and Virla 2014); and in Argentina 47 (Olmi and Virla 2014). The higher numbers of *Gonatopus* species from Brazil and Argentina suggest that the true number of species in Mexico will ultimately be much higher. Further research, also on the hosts, will be needed to better characterise this fauna. In fact, hosts are known only for 12 of the 26 *Gonatopus* species recorded from Mexico (Becerra-Chiron et al. 2017, Guglielmino et al. 2013): another gap to be bridged, in spite of the contributions of Prof Moya-Raygoza and his research group (Moya-Raygoza and Olmi 2010, Becerra-Chiron et al. 2017). Among these 12 hosts, leafhopper pests of maize in the Neotropical region are economically important (Guglielmino et al. 2006).

## Supplementary Material

XML Treatment for Gonatopus

XML Treatment for Gonatopus jaliscanus
